# Kognitive Beeinträchtigungen bei schizophrenen Psychosen: Diagnostik, Verlauf und Therapie

**DOI:** 10.1007/s00115-024-01773-8

**Published:** 2024-11-26

**Authors:** Lana Kambeitz-Ilankovic, Wolfgang Strube, Bernhard T. Baune, Peter Falkai, Lukas Röll, Stefan Leucht

**Affiliations:** 1 Klinik und Poliklinik für Psychiatrie und Psychotherapie, Universitätsklinikum Köln, Köln, Deutschland; 2 Klinik für Psychiatrie, Psychotherapie und Psychosomatik, Medizinische Fakultät, Universität Augsburg, Augsburg, Deutschland; 3 DZPG (Deutsches Zentrum für Psychische Gesundheit), Standort München/Augsburg, München, Deutschland; 4 Klinik für Psychische Gesundheit, Universitätsklinikum Münster, Münster, Deutschland; 5 Department of Psychiatry, University of Melbourne, Melbourne, Australien; 6 Florey Institute of Neuroscience and Mental Health, Parkville, Melbourne, Australien; 7 Klinik für Psychiatrie und Psychotherapie, LMU Klinikum München, München, Deutschland; 8 Max-Planck-Institut für Psychiatrie, München, Deutschland; 9 Klinik und Poliklinik für Psychiatrie und Psychotherapie, Technische Universität München, TUM School of Medicine and Health, Ismaninger Straße 22, 81675 München, Deutschland

**Keywords:** Psychose, Kognitive Leistung, Antipsychotika, Psychosoziale Interventionen, Psychosoziales Funktionsniveau, Psychosis, Cognitive performance, Antipsychotics, Psychosocial interventions, Psychosocial functioning

## Abstract

**Hintergrund:**

Längsschnittstudien zeigen, dass die Mehrzahl der Betroffenen mit schizophrenen Psychosen schizophrenieassoziierte kognitive Defizite (CIAS) entwickelt.

**Ziel der Arbeit:**

Überblick über Epidemiologie, Diagnostik und Evidenz verschiedener Therapieoptionen von CIAS.

**Material und Methoden:**

Übersicht aktueller Befunde zur Wirksamkeit verschiedener Therapien bei CIAS.

**Ergebnisse:**

Bis zu 85 % der Betroffenen mit schizophrenen Psychosen zeigen CIAS, z. T. bereits vor der Entwicklung von Positiv- oder Negativsymptomen. CIAS gehen mit einer hohen individuellen Belastung einher, weil viele alltagsrelevante Bereiche des Funktionsniveaus beeinträchtigt sind. Zur klinischen Erfassung stehen verschiedene Testbatterien zur Verfügung. Als Standard für Studien und spezielle klinische Fragestellungen gilt die MATRICS Consensus Cognitive Battery (MCCB). Die Behandlung von CIAS erfordert ein multimodales Vorgehen, wobei aktuell nichtmedikamentöse Strategien (z. B. kognitive Remediation, Sporttherapie) die beste Evidenz vorzuweisen haben. Nichtinvasive Neurostimulationsverfahren und dopaminerge Antipsychotika der 1. und 2. Generation wirken kaum auf kognitive Funktionsstörungen im Rahmen schizophrener Psychosen.

**Diskussion:**

CIAS ist ein häufiges, krankheitsimmanentes Symptom bei schizophrenen Psychosen, das im klinischen Alltag beachtet werden sollte, da es die Betroffenen erheblich in ihrem Funktionsniveau und ihrer Lebensqualität beeinträchtigt. Therapeutische Optionen sind limitiert, doch zeigen innovative psychosoziale Interventionen kleine bis moderate Effekte. Zudem könnten neue, auf Basis aktueller neurobiologischer Erkenntnisse entwickelte Medikamente sowie Kombinationen mit psychosozialen und Neurostimulationsverfahren Perspektiven eröffnen.

## Abstract (en)

**Background:**

Longitudinal outcome studies confirm that the majority of patients with schizophrenic psychoses develop cognitive impairments associated with schizophrenia (CIAS).

**Objective:**

To provide an overview of the epidemiology, diagnostics and evidence for various treatment options for CIAS.

**Material and methods:**

Literature review of the current level of evidence regarding the efficacy of different treatment strategies for CIAS.

**Results:**

Up to 85% of patients with schizophrenic psychoses exhibit CIAS, in some cases even before the development of positive or negative symptoms. The CIAS are associated with extensive individual burden due to impairments in many areas of cognitive and psychosocial functioning relevant to daily life. Various test instruments are available for clinical assessment with the Mental Health’s Measurement and Treatment Research to Improve Cognition in Schizophrenia (MATRICS) consensus cognitive battery (MCCB) as an established standard for clinical trials and special clinical issues. The treatment of CIAS warrants a multimodal approach with non-drug strategies (e.g., cognitive remediation, exercise) currently providing the best level of evidence. Noninvasive neurostimulation procedures and dopaminergic antipsychotic drugs of the first and second generations have demonstrated low effectiveness on cognitive function disorders in schizophrenic psychoses.

**Conclusion:**

The CIAS is a frequent disease-immanent symptom in schizophrenic psychoses that should be considered in the clinical routine as it substantially impairs those affected in the functional level and quality of life. Current treatment options are limited but innovative psychosocial interventions show low to moderate effects. In addition, new medications developed based on current neurobiological findings and combinations with psychosocial and neurostimulation procedures could open up new perspectives.

Kognitive Funktionsstörungen zählen zu den unterschätzten Symptomen der Schizophrenie. Wegen der Assoziation zwischen kognitiven Störungen und sozialem Funktionsniveau wirken sie sich unmittelbar auf die Lebensqualität aus, vermutlich stärker als andere krankheitsbezogene Faktoren [12]. Die Therapie sollte multimodal erfolgen, wobei kognitive Remediation, Sporttherapie und Neurostimulation insbesondere in Kombination als vielversprechend diskutiert werden. Bisher sind die pharmakotherapeutischen Optionen unzureichend, aber aktuelle neurobiologische Erkenntnisse lassen auf spezifisch wirksame Medikamente hoffen.

## Definition und Epidemiologie von CIAS

Schizophrenie ist eine der schwerwiegendsten psychischen Erkrankungen mit der höchsten kognitiven Beeinträchtigung [17]. Am häufigsten werden in diesem Kontext Störungen von Aufmerksamkeit und Vigilanz, Arbeitsgedächtnis, exekutiven Funktionen, episodischem und verbalem Gedächtnis, sozialer Kognition und Sprachverarbeitung diskutiert [17]. In geringerem Maße wurden auch Beeinträchtigungen des semantischen oder visuellen Gedächtnisses, der Verarbeitungsgeschwindigkeit und des prozeduralen Gedächtnisses festgestellt [17]. Damit gehören zu den Symptombereichen der Schizophrenie neben Positivsymptomen (Halluzinationen, Wahnvorstellungen) und Negativsymptomen (Avolition, Alogie, Autismus, Anhedonie, abgestumpfter Affekt, soziale Vermeidung) auch Einschränkungen der Kognition. Die Gesamtheit kognitiver Symptome im Zusammenhang mit Schizophrenie wird als „cognitive impairment associated with schizophrenia“ bezeichnet, kurz „CIAS“. In Längsschnittstudien litten bis zu 85 % aller Schizophreniepatienten an einer kognitiven Beeinträchtigung [26].

## CIAS beeinflusst den Krankheitsverlauf

Die wiederholte Beobachtung, dass die Kognition bereits vor dem Auftreten von Positiv- oder Negativsymptomen beeinträchtigt sein kann, nährt die Annahme, dass CIAS als krankheitsimmanent anzusehen ist. Anscheinend sagen Einbußen bei Verarbeitungsgeschwindigkeit, verbalem Gedächtnis und sozialer Kognition bei Hochrisikopersonen die Umwandlung in eine vollständige Psychose voraus [1]. Sozialer Rückzug infolge beeinträchtigter sozialer Kognition verschlimmert die negativen Symptome [17]. Ermutigend ist, dass bei guter Symptomkontrolle und stabilem Krankheitsverlauf zumindest einige Teilbereiche der Kognition stabil bleiben oder sich sogar im Verlauf der Erkrankung verbessern können [26]. Dies steht im Einklang mit der Beobachtung, dass Patienten mit einer ersten Episode kognitiv schlechter abschneiden als Patienten, die vor 2 Jahren oder länger eine erste Psychose erlebt haben und mittels medikamentöser Behandlung im Anschluss eine längere stabile Phase erreichen konnten (durchschnittliche Erkrankungsdauer von 80 Monaten). Das Ausbleiben akuter Episoden über einen längeren Zeitraum oder das Fehlen vorherrschender negativer oder desorganisierter Symptome sind in diesem Zusammenhang entscheidend [26]. Bislang gibt es keine Hinweise auf geschlechtsspezifische Unterschiede.

## Methoden zur Erfassung von CIAS

Bei der klinischen Erfassung von CIAS stehen sieben Bereiche im Mittelpunkt: Arbeitsgedächtnis, Aufmerksamkeit und Vigilanz, verbales Lernen und Gedächtnis, visuelles Lernen und Gedächtnis, logisches Denken und Problemlösen, Verarbeitungsgeschwindigkeit und soziale Kognition. Alle diese Domänen sind in der MATRICS Consensus Cognitive Battery (MCCB) vertreten (Tab. 1), die aus einem Expertenkonsens als Methode zur gründlichen Prüfung der CIAS hervorgegangen ist [5]. Die BACS (Brief Assessment of Cognition in Schizophrenia) wird als wertvolle Alternative angesehen, da sie sich auf sechs kognitive Bereiche konzentriert, dafür jedoch nur 35 min benötigt statt 70–90 min wie die MCCB. Alternativ können einzelne Funktionen (z. B. exekutive Funktionen) geprüft werden. Aufgrund des potenziellen prognostischen Werts von CIAS sollten auch Screeningmethoden wie SCIP (Screen for Cognitive Impairment in Psychiatry), ein 15-minütiger Test, der Fälle von Kontrollen unterscheidet, und B‑CATS (Brief-Cognitive Assessment Tool for Schizophrenia), ein 10-minütiger Test mit guter Korrelation zum MATRICS, in Betracht gezogen werden, um Risikopersonen zu identifizieren. Die SCoRS (Schizophrenia Cognition Rating Scale) schließlich ist ein interviewbasiertes Screeninginstrument, das die kognitiven Bereiche in Bezug auf ihre reale, alltägliche Funktionalität testet [11].

**Tab. 1:** MATRICS Consensus Cognitive Battery (MCCB). (Mod. [5])

Kognitive Domäne MATRICS	Verwendete Tests	Leistungsbasierte Bewertung/Empfehlung
Verarbeitungsgeschwindigkeit	Category Fluency BACS Symbolerkennung Trail Making Test A	*Zeit:* ca. 60–90 min (Pausen erlaubt) *Score* bei gesunden Freiwilligen: 50 ± 10 *Validierung und Richtlinien*: u. a. von Europäische Arzneimittel-Agentur (EMA) und Food and Drug Administration (FDA) als primärer Endpunkt für CIAS akzeptiert (European Psychiatric Association (EPA)-Konsens)
Aufmerksamkeit/Vigilanz	Continuous Performance Test („identical pairs version“)	
Arbeitsgedächtnis (nichtverbal und verbal)	Letter-number Span Test WMS-III Spatial Span Test	
Verbales Lernen	Hopkins Verbales Lernen‑R	
Visuelles Lernen	Kurzer Visuospatial Test‑R	
Logisches Denken und Problemlösung	NAB mazes	
Soziale Kognition	MSCEIT Test	
**Kognitive Domäne B‑CAT**		
Ein globaler Kognitionswert B‑CATS ist nicht dazu gedacht, die kognitive Funktion auf der Ebene der Domänen zu testen	Category Fluency BACS Symbolerkennung Trail Making Test B	*Zeit: *ca. 10–20 min *Validierung und Richtlinien: *Er korreliert in hohem Maße mit den Gesamtergebnissen umfassender neuropsychologischer Test (0,73–0,86) *Administration****:*** keine spezialisierte neuropsychologische Ausbildung ist notwendig

MCCB testet 7 kognitive Domänen, die funktionell signifikante Bereiche der Beeinträchtigung bei schizophrenen Psychosen darstellen und sich möglicherweise für eine pharmakologische Behandlung eignen

*MSCEIT Test* The Mayer-Salovey-Caruso Emotional Intelligence Test, *NAB* Neuropsychological Test Battery, *WMS-III* Wechsler Memory Scale

Die European Psychiatric Association plädiert für eine bessere, einheitliche Erfassung von CIAS, sowohl in Form von Selbstauskünften als auch in Form von Tests. Diese Empfehlung zielt auf eine konsequente, standardisierte Methodik zur Erfassung von CIAS bei Patienten in allen Phasen der Störung, auch bei Personen mit einem erhöhten Risiko, eine Psychose zu entwickeln. Darüber hinaus sollte eine klinische Erfassung mit direkten Fragen zu kognitiven Fähigkeiten zum Standard der klinischen Anamnese gehören. Simple Fragen zu Alltagstätigkeiten wie Informationsaufnahme beim Lesen, Fernsehen, Onlinelesen, Organisation von Einkäufen, Durchführung von Bankgeschäften und Haushaltstätigkeiten können hilfreiche Hinweise auf kognitive Einschränkungen geben.

## Neurobiologische Grundlagen der Kognition

**Abb. 1:**
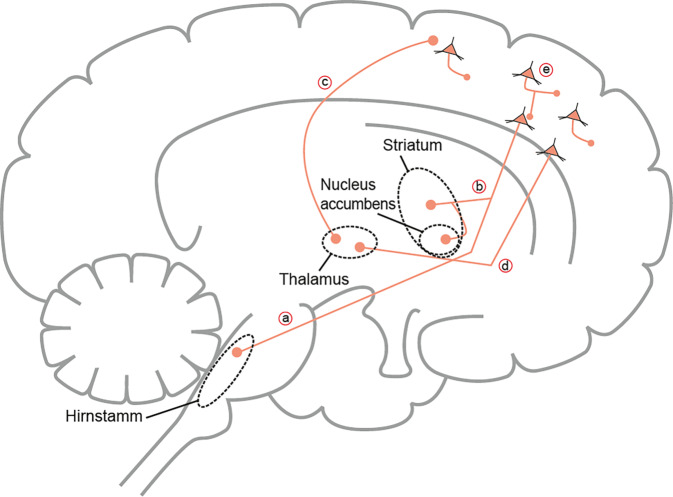
Viele Hirnschaltkreise, die eine Schlüsselrolle bei der Regulation kognitiver Prozesse und Funktionen spielen, unterliegen einer glutamatergen Neurotransmission. Relevant sind v. a. glutamaterge Bahnen vom präfrontalen Kortex zu tieferliegenden Hirnstammregionen (*a*), zum Striatum (*b*) und zum Thalamus (*c*, *d*). Kortikale Pyramidalneurone (*e*) sind ebenfalls glutamaterg verschaltet. (Mod. nach [23])

In den letzten Jahren konnten wichtige Fortschritte auf dem Gebiet der neurobiologischen Forschung erzielt werden, die das Verständnis kognitiver Prozesse vertiefen. Der durch den Neurotransmitter Glutamat aktivierte N‑Methyl-D-Aspartat-Rezeptor (NMDAR) spielt dabei eine bedeutsame Rolle [22]. Glutamat ist in exzitatorische, der Neurotransmitter γ‑Aminobuttersäure (GABA) in inhibitorische neuronale Prozesse eingebunden, welche eine Vielzahl an neuronalen Funktionen wie Lernen und Gedächtnis, neuronale Netzwerkfunktionen und synaptische Plastizität unterstützen. Hinsichtlich der kognitiven Domänen Arbeitsgedächtnis, Exekutivfunktionen und episodisches Gedächtnis sind u. a. die glutamaterge Transmission im präfrontalen Kortex (PFC) oder Hippokampus sowie verschiedene Hirnschaltkreise innerhalb des PFC zum ventralen Hippokampus und zum ventralen Striatum eingebunden (Abb. 1; [23]).

Der Glutamathypothese zufolge werden klinische Symptome der Schizophrenie wie z. B. kognitive Einschränkungen u. a. durch eine prolongierte Hypofunktion am NMDAR verursacht. CIAS geht mit einer reduzierten Aktivierung oder Funktion von NMDA-Rezeptoren auf GABAergen inhibitorischen Interneuronen in Hirnregionen wie dem PFC einher, was zu Defiziten in synaptischer Plastizität und kortikalen Netzwerken (exzitatorische/inhibitorische Balance) und somit kognitiven Beeinträchtigungen führen kann [22].

## Antipsychotika ohne überzeugenden Effekt auf CIAS

Die hochpotenten Antipsychotika der 1. Generation bewirken überwiegend eine Dopaminblockade. Durch diese lassen sich Positivsymptome reduzieren, Effekte auf Negativsymptome sind schwächer ausgeprägt. Da dopaminerge Neurotransmission für kognitive Funktionen wesentlich ist, kann eine Dopaminblockade diesbezüglich ungünstig sein. Die Antipsychotika der 2. Generation haben die in sie gesetzten Erwartungen hinsichtlich der Wirksamkeit und der Nebenwirkungsreduktion bestenfalls zum Teil erfüllt, auch im Hinblick auf CIAS [20]*.*

Zu den Effekten auf kognitive Funktionen zeigte schon 2004 eine Publikation, dass auch Antipsychotika der 1. Generation kognitive Funktionen im Vergleich zu Placebo im Mittel bei Schizophrenie verbessern, doch die Effektstärke war sehr klein [18]. Auch ist unklar, ob es sich um eine Besserung kognitiver Funktionen wie Arbeitsgedächtnis usw. handelt oder nur um eine indirekte Verbesserung durch Minderung formaler Denkstörungen. Diese Überlegung würde allerdings auch für jede andere medikamentöse Intervention gelten. Bei erneuter Verschlechterung der Psychose könnte es sein, dass die dazugehörenden formalen Denkstörungen sich insbesondere in der Placebogruppe verschlechtern und aus diesem Grund die kognitiven Einbußen in der Verumgruppe unter z. B. Haloperidol kleiner ausfallen. Dies gilt ebenso für Placebovergleiche. Allerdings ist es eher unwahrscheinlich, dass in den Studien ein erheblicher Anteil der Patienten eine Verschlechterung der formalen Denkstörungen im Lauf der Studienphase erlitten hat, weil der „The Positive and Negative Syndrome Scale“(PANSS)-Gesamtwert und die Positivskala während des Studienverlaufs abnahmen.

Bei Vergleichen von Antipsychotika der 1. und 2. Generation ergaben einfache paarweise Metaanalysen zunächst eine geringfügige Überlegenheit der neuen Medikamente, Effektstärke etwa 0,2 [30]. Allerdings diente hier als Vergleichsmedikament Haloperidol, ein starker Dopaminblocker, und es wurde in den Studien in heute (vor allem in der längerfristigen Therapie) nicht mehr üblichen hohen Dosierungen von bis zu 20 mg pro Tag eingesetzt. Dopamin ist wichtig für zahlreiche kognitive Funktionen. Daher ist die Verwendung starker Dopaminblocker a priori ungünstig. Der Dopaminantagonist Perphenazin schnitt am Ende (18 Monate) der großen industrieunabhängigen „Clinical Antipsychotic Trials of Intervention Effectiveness“(CATIE)-Studie (1493 Teilnehmer) hinsichtlich der Kognition signifikant besser als die Zweitgenerationsmedikamente Olanzapin, Risperidon, Quetiapin und Ziprasidon ab [10]. Allerdings waren nur knapp 21 % der Patienten so lange in der Studie geblieben. Nach 2 Monaten fand sich kein Unterschied zwischen den Medikamenten. Clozapin als eines der potentesten atypischen Antipsychotika der 2. Generation, wirkt sedierend und stark anticholinerg und daher ungünstig auf die kognitive Leistungsfähigkeit.

Es folgten methodisch aufwendigere Netzwerkmetaanalysen, die jedoch heterogene Resultate brachten. Eine Analyse fand z. B. nur einzelne günstige Effekte etwa von Amisulprid auf verbales Lernen oder von Perphenazin auf exekutive Funktion. In der neuesten Arbeit schlossen einerseits Haloperidol und Fluphenazin, andererseits Clozapin eher schlecht ab [3]. Diese Präparate sollte man daher mit Blick auf das Ziel einer positiven Modifizierung von CIAS eher vermeiden.

Eine europäische Konsensusleitlinie diskutiert weitere Medikamente und Substanzklassen wie Antidepressiva, N‑Acetylcystein, D‑Serin, Acetylcholinesteraseinhibitoren, Oxytocin, Cannabidiol und antiinflammatorische Substanzen. Sie empfiehlt letztlich aber neben Antipsychotika der 2. Generation nur das Vermeiden anticholinerger Substanzen und von Benzodiazepinen [28]. Insgesamt gibt es aktuell also keine medikamentösen Strategien, die zur Verbesserung von CIAS empfohlen werden können.

## Neue Ansätze orientieren sich an der Neurobiologie

Derzeit befindet sich eine Reihe innovativer Ansätze zur medikamentösen Behandlung von CIAS in der klinischen Entwicklung. Ihnen ist gemeinsam, dass sie nicht am dopaminergen System wirken, sondern über andere für die Kognition relevante Transmittersysteme. Einige davon sollen im Folgenden kurz beschrieben werden.

Der selektive Glyzin-Transportersystem1(GlyT1)-Inhibitor Iclepertin verstärkt die glutamaterge Signalübertragung, indem er die Verfügbarkeit des als NMDA-Rezeptor-Koaktivator wirksamen Glyzins im synaptischen Spalt erhöht. Nach positiven Ergebnissen der Phase-2-Studie wird Iclepertin aktuell im Phase-3-Studienprogramm CONNEX untersucht [22].

Wie Iclepertin soll Luvadaxistat als D‑Aminosäureoxidase-Inhibitor das Signal am NMDAR verstärken. Der Wirkmechanismus basiert darauf, dass es die Verfügbarkeit des NMDA-Koagonisten D‑Serin an zentralnervösen Synapsen erhöht. Nach Abschluss der präklinischen Untersuchungen wird auch dieser Wirkstoff jetzt klinisch erprobt [21].

Ein innovativer muskarinerger Ansatz wird mit dem Kombinationspräparat KarXT verfolgt, das an den zentralen muskarinischen Acetylcholin(mACh)-Rezeptoren wirkt. Es vereint den Muskarinagonisten Xanomelin, der bevorzugt die zentralen M1/M4-Rezeptoren stimuliert, und den peripher wirksamen Muskarinantagonisten Trospiumchlorid. Durch die Kombination sollen die über die Aktivierung peripherer mACh-Rezeptoren vermittelten Nebenwirkungen reduziert werden. Im laufenden Studienprogramm EMERGENT konnten neben einer Verbesserung der Positiv- sowie Negativsymptomatik zuletzt auch Effekte auf die Kognition dargestellt werden [15]. Auch die Entwicklungssubstanz Emraclidin folgt einem nichtdopaminergen Ansatz durch Aktivierung der zentralen muskarinergen M4-Rezeptoren, was alle Symptomdomänen der Schizophrenie günstig beeinflussen soll [14].

## Kognitive Remediation verbessert Alltagsfunktionen

Kognitive Remediation (KR) ist eine der relevantesten Therapien zur Verbesserung kognitiver Defizite. Als eine auf Verhaltenstraining basierende Intervention zielt KR darauf ab, kognitive Prozesse wie Aufmerksamkeit, Gedächtnis, exekutive Funktionen und soziale Kognition durch angeleitetes, wiederholtes Üben einer Reihe standardisierter und verhaltensbasierter Aufgaben und Strategien zu steigern [31]. Diese spezifischen Lernereignisse sollen gleichzeitig kognitive Funktionen sowie deren zugrunde liegende Abläufe im neuronalen System (z. B. Neuroplastizität) verbessern [27].

Dabei kommen unterschiedliche Trainingsmodalitäten infrage wie Stift und Papier, computergestütztes oder durch Menschen angeleitetes Training, die nach Bedarf kombiniert werden können. Die aktuelle Metaanalyse berichtete über geringe bis mäßige Effekte von KR (*d*, 0,29), insbesondere bei verbalem Gedächtnis (*d*, 0,25), Arbeits- (*d*, 0,25) und visuellem Gedächtnis (*d*, 0,25) sowie den exekutiven (*d*, 0,28) und sozialen kognitive Funktionen (*d*, 0,24). Damit ist KR bereits fester Bestandteil der S3-Therapieleitlinie (Empfehlungsgrad A). Neben der direkten Verbesserung der kognitiven Funktionen stellt die Wiederherstellung der Alltagsfunktionen ein wichtiges Ziel der KR dar, da Ausbruch und Verlauf einer Psychose mit schwerwiegenden sozialen Folgen wie sozialer Isolation und beruflichen Schwierigkeiten verbunden sind [9]. In mehreren Metaanalysen waren die Effekte von KR auf Kognition und Funktionsniveau bewiesen (*d, *0,22), insbesondere das berufliche Funktionsniveau, wenn sie mit psychiatrischer Rehabilitation oder zusätzlicher menschlicher Betreuung kombiniert wurden [28]. 

## KR findet bei Patienten hohe Akzeptanz

Systematische Untersuchungen der Durchführbarkeit von KR bei Schizophrenie zeigten eine hohe Akzeptanz der Intervention. In Studien entsprach die Abbruchrate bei Patienten, die KR erhielten (16,58 %), der Abbruchrate bei Kontrollinterventionen (15,21 %) und war vergleichbar mit der Abbruchrate verschiedener psychosozialer Interventionen für Personen mit diagnostizierter Schizophrenie [29]. Interessanterweise scheint die Akzeptanz von KR höher in Studien mit stationären Patienten sowie bei Patienten mit niedrigerem IQ und niedrigerem Ausbildungsniveau. Was die Dauerhaftigkeit der kognitiven Verbesserungen betrifft, so gibt es Hinweise, dass KR in der Lage ist, bei Schizophrenie langfristige Vorteile zu erzielen. In einer 1‑jährigen Follow-up-Studie wurden anhaltende Verbesserungen in multiplen kognitiven Bereichen festgestellt sowie Verbesserungen in der sozialen und täglichen Funktionsfähigkeit. Auf neuroanatomischer Ebene deuten Studien im Früh- und Spätstadium der Psychose darauf hin, dass die KR neuroplastische bzw. neuroprotektive Veränderungen im medialen präfrontalen Kortex, Temporallappen, Hippokampus und limbischen Kortex hervorruft, die mit einer Verhaltensverbesserung einhergehen [2]. Neue Studien zur KR haben sich mit der Heterogenität der Trainingsreaktion befasst und Verfahren aus dem Bereich der künstlichen Intelligenz (KI) eingesetzt, um individuelle Prädiktoren für das Therapieansprechen zu identifizieren wie z. B. strukturelle und funktionelle Gehirnmuster sowie spezifische Profile des Lernverhaltens.

## Sporttherapien gegen kognitive Defizite

Sport hat sich als Zusatztherapie bei vielen chronischen Erkrankungen in und außerhalb der Psychiatrie bewährt. Auch bei Menschen mit Schizophrenie konnten in zahlreichen großen Metaanalysen die positiven Effekte verschiedener Arten von Sporttherapien auf allgemeine Symptomschwere (z. B. [16]), Positiv- und Negativsymptomatik bei Schizophrenie gezeigt werden. Zwischen 2017 und 2023 wurden ferner drei Metaanalysen und eine große randomisierte kontrollierte Multicenterstudie zu den Auswirkungen von Sporttherapien auf kognitive Defizite bei Menschen mit Schizophrenie veröffentlicht [4, 16, 25, 32]. Zu den Studienpopulationen gehörten Patienten in gänzlich unterschiedlichen Stadien der Erkrankung und mit unterschiedlicher Krankheitsschwere. Die meisten Originalstudien untersuchten speziell den aeroben Ausdauersport als zentrale sportbezogene Therapieform, jedoch flossen auch Erkenntnisse bezüglich Kraft- und Beweglichkeitstrainings oder Yoga in die Analysen ein. Sporttherapien zeigten im Vergleich zu aktiven oder passiven Kontrollbedingungen positive Effekte auf die allgemeine kognitive Leistungsfähigkeit bei Menschen mit Schizophrenie [4, 25, 32]. Dabei schwanken die Effektgrößen zwischen 0,21 und 0,33 (Tab. 2), was einem kleinen positiven Effekt entspricht. Der aktuelle Forschungsstand verdeutlicht also die allgemeine Wirksamkeit von (aeroben) Sporttherapien, um kognitive Funktionen bei Menschen mit Schizophrenie zu verbessern.

**Tab. 2:** Effekte von Sporttherapie auf die Kognition

Typ	Art der Sporttherapie	Primärer Endpunkt	Zahl der Studien/Probanden	Effektgröße	Autor, Jahr
Metaanalyse und Review	Aerober Sport, Yoga und andere	Kognition	10 Studien	Hedges’ g = 0,33	Firth et al. 2016 [4]
Review	Aerober Sport	Kognition	15 Studien	SMD = 0,21	Shimada et al. 2022 [25]
Metaanalyse	Aerober Sport	Kognition	5 Studien	SMD = 0,31	Xu et al. 2022 [32]
Randomisierte kontrollierte Studie	Aerober Sport, Kraft‑/Beweglichkeitstraining	Kognition	92 Teilnehmer	SMD = 0,21	Maurus et al. 2023 [16]

*SMD* „standardized mean difference“

## Wirksam und praktisch nebenwirkungsfrei

Es stellt sich die Frage, ob der kleine, aber statistisch robuste Effekt von Sporttherapien auf die allgemeine kognitive Leistungsfähigkeit von Menschen mit Schizophrenie klinisch relevant ist. Die beobachteten Effektgrößen von Sporttherapien sind mit denen kognitiver Trainingsprogramme vergleichbar, jedoch aus kleineren Stichproben abgeleitet. Zur klinischen Relevanz dieser Effekte konnten gezeigt werden, dass kognitive Verbesserungen als Folge kognitiver Trainingsprogramme das allgemeine Funktionsniveau im Alltag der Patienten steigern [9]. Drei Metaanalysen zu Sporttherapien zeigen zudem positive Effekte auf das allgemeine Funktionsniveau bei Schizophrenie. Daraus lässt sich ableiten, dass die prokognitiven Effekte von Sporttherapien bei Schizophrenie höchstwahrscheinlich klinisch relevant sind. Es gibt zudem Anhaltspunkte, dass sich die Effekte von Sporttherapien auf Subdomänen der Kognition unterscheiden. Allerdings sind die Resultate der Metaanalysen hier uneindeutig [4, 25, 32]. Ein umfassender Vergleich der Wirksamkeit verschiedener Sporttherapien bleibt herausfordernd, da die meisten Metaanalysen aerobe Trainingsprogramme in den Blick nahmen [4, 25, 32]. Aktuell ist anzunehmen, dass die Unterschiede marginal sind. Jedenfalls zeigt eine aktuelle randomisierte kontrollierte Multicenterstudie, dass sowohl aerober Ausdauersport als auch Kraft- und Beweglichkeitstraining positive Auswirkungen auf die globale kognitive Funktion bei Schizophrenie haben [13].

Der aktuelle Forschungsstand liefert erste Einblicke in Dosis-Wirkungs-Beziehungen von Sporttherapien zur Behandlung kognitiver Defizite bei Schizophrenie. Es wird angenommen, dass Sporttherapien mit moderater Intensität für mindestens 90 min pro Woche über mindestens 12 Wochen erforderlich sind, um eine optimale Wirkung zu erzielen. Dies kann ein aerobes Ausdauertraining zu Fuß (Joggen, Nordic Walking etc.) oder auf dem Fahrrad sein. Je nach Vorliebe der Patienten kann jedoch auch ein Kraft- und Beweglichkeitsprogramm durchgeführt werden. Dieses Training sollte in Kleingruppen mit anderen Betroffenen und unter professioneller Supervision durch entsprechendes Fachpersonal absolviert werden. Hierfür sollten behandelnde Kliniken Kooperationen mit lokalen Fitnessstudios, Sportvereinen, Sportgruppen oder Ähnlichem aufbauen, um Patienten nach stationärem Aufenthalt dort direkt anbinden zu können. Eine professionelle Betreuung der Therapieeinheiten und ein Gruppensetting mit anderen Betroffenen erhöht die Wirksamkeit [4, 25, 32]. Zugleich können Sporttherapien als sicher und praktisch nebenwirkungsfrei angesehen werden, jedoch wird vor dem Beginn der Therapie eine Untersuchung der Sporttauglichkeit der Patienten empfohlen.

## Nichtinvasive Neurostimulation wird geprüft

In den letzten Jahren wird verstärkt untersucht, inwieweit nichtinvasive Verfahren zur Neuromodulation („non-invasive brain stimulation“, NIBS) auch genutzt werden können, um kognitive Einschränkungen bei Menschen mit Schizophrenie zu modifizieren. Dabei ist vor allem die repetitive transkranielle Magnetstimulation (rTMS) von Interesse, da deren Wirksamkeit hinsichtlich kognitiver Funktionen bei anderen psychischen Erkrankungen positiv diskutiert wird [8]. Bei der rTMS-Behandlung werden repetitive Reizserien magnetischer Impulse genutzt, um neuroplastische Prozesse anzuregen. Ein Behandlungszyklus umfasst wochentägliche Sitzungen von 5–30 min Dauer über 4 bis 6 Wochen – je nach Stimulationsprotokoll. Systematische Übersichtsarbeiten über randomisierte kontrollierte Studien konnten bisher nur geringe Effekte auf unterschiedliche kognitive Domänen nachweisen [6, 24]. Auch eine Metaanalyse berichtete nur geringe oder fehlende Effektstärken der rTMS-Behandlung auf unterschiedliche kognitive Domänen, wobei hinsichtlich Arbeitsgedächtnis und Sprachverarbeitung geringe, jedoch anhaltende Effekte festgestellt wurden [7]. Derzeit besteht noch eine deutliche Heterogenität zwischen den Studien in Bezug auf die Einschlusskriterien, verwendete Stimulationsparameter, angewandte kognitive Messverfahren und Nachbeobachtungsintervalle. Dies und Barrieren hinsichtlich der Implementierung von rTMS in die Versorgungslandschaft außerhalb spezialisierter Zentren schränken derzeit eine Übertragbarkeit in einen klinischen Behandlungskontext ein.

## Gleichstromstimulation möglicherweise vielversprechender

Die transkranielle Gleichstromstimulation („transcranial direct current stimulation“, tDCS) stellt demgegenüber eine weitere nichtinvasive Methode zur Neuromodulation dar, die sich einfach, auch mittels portabler Geräte, in die (ambulante) Versorgung von Menschen mit CIAS integrieren ließe. Bei der tDCS werden Hautelektroden dazu genutzt, schwache Gleichstromreize im Milliamperebereich zu erzeugen, um neuronale Plastizität zu fördern. Eine klinische Pilotstudie konnte bei Add-on-Behandlung mit tDCS zusätzlich zu einer Behandlung mit KR einen augmentativen Effekt nachweisen, wobei derzeit noch bestätigende Untersuchungen ausstehen. Angesichts der möglichen augmentativen Effekte konzentrieren sich neue Forschungsbemühungen auf die Integration nichtinvasiver Neurostimulationsverfahren in Komplexbehandlungen zusätzlich zu pharmakologischen und nichtpharmakologischen Strategien. Die Ergebnisse von systematischen Übersichtsarbeiten und Reviews deuten dabei darauf hin, dass tDCS die Verarbeitungsgeschwindigkeit, die Exekutivfunktionen und insbesondere das Arbeitsgedächtnis bei Menschen mit einer Schizophrenie verbessern könnte [19]. Aufgrund der methodischen Heterogenität der bisher vorliegenden Studien kann jedoch noch keine Empfehlung geeigneter Protokolle erfolgen. Vielmehr ist die Entwicklung standardisierter Stimulationsprotokolle für die klinische Praxis Gegenstand weiterer Forschungsbemühungen.

Zusammenfassend stellen rTMS und tDCS-Verfahren dar, die das Potenzial besitzen könnten, bei bestimmten Subpopulationen Betroffener mit CIAS pharmakologische und nichtpharmakologische Intervention zur Verbesserung bestimmter kognitiver Funktionen wesentlich zu unterstützen.

## Fazit für die Praxis

- Da CIAS zu den häufigsten Begleitsymptomen schizophrener Psychosen zählt und viele alltagsrelevante Fähigkeiten beeinträchtigt, sollten Patienten routinemäßig befragt und mit standardisierten Tests wie MCCB oder BACS untersucht werden, ob kognitive Funktionseinschränkungen vorliegen.
- Medikamentöse Therapien haben bislang keine überzeugenden Effekte auf CIAS gezeigt. Empfohlen wird, zur Pharmakotherapie der Psychose vorzugsweise Arzneistoffe ohne ausgeprägte anticholinerge Wirkung einzusetzen, um ungünstige Effekte auf die Kognition zu vermeiden. Neue, an der Neurobiologie von CIAS orientierte Wirkstoffe befinden sich in Prüfung.
- Nichtmedikamentöse Therapiestrategien können sich auf ausreichende Evidenz stützen. Betroffene profitieren vor allem von kognitiver Remediation und Sporttherapien.
- Neurostimulationsverfahren erreichen bislang nur geringe Wirksamkeit, wobei kombinierte Ansätze (z. B. in Kombination mit kognitiver Remediation) möglicherweise bessere Effekte erzielen.
- Neue Studien aus dem Bereich der künstlichen Intelligenz (KI) untersuchen, welche Kombination der genannten Therapien für individuelle Patienten geeignet ist, indem sie versuchen, Prädiktoren für das Therapieansprechen zu identifizieren wie z. B. strukturelle und funktionelle Gehirnmuster sowie spezifische Profile der demografischen und klinischen Charakteristika.
- Bisher sind die Befunde uneindeutig, da sie auf kleinen Kollektiven von Patientendaten basieren. Künftige KI-Studien lassen hoffen, in Zusammenarbeit mit dem Kliniker die optimale Entscheidung in Bezug auf eine personalisierte Therapie zu treffen.
